# Integrating Developmental Biology with Population Health

**DOI:** 10.3390/life16071040

**Published:** 2026-06-23

**Authors:** Paschalis Theotokis, Soultana Meditskou, Maria Eleni Manthou

**Affiliations:** Department of Histology-Embryology, School of Medicine, Aristotle University of Thessaloniki, 54124 Thessaloniki, Greece; ptheotokis@auth.gr (P.T.); sefthym@auth.gr (S.M.)

Human development is shaped by a complex interplay of molecular regulation, embryonic patterning, maternal–fetal interactions, environmental influences, and population-level determinants. The Special Issue “From Stem Cells to Embryos, Congenital Anomalies and Epidemiology” brings together contributions that collectively explore this multidimensional landscape, spanning stem cell biology, prenatal development, congenital anomalies, neonatal health, and epidemiological surveillance. By integrating mechanistic and clinical perspectives, the studies included in this collection emphasize the importance of understanding development not only at the cellular and embryological level, but also within broader physiological, environmental, and public health contexts.

A central theme emerging from this Special Issue is the role of stem cells and regenerative approaches in reproductive and developmental medicine. Mesenchymal stem cells (MSCs) are explored as promising therapeutic tools in female infertility, reflecting the growing interest in many regenerative strategies capable of restoring reproductive function and improving fertility outcomes [1]. These advances highlight the translational potential of stem cell biology while also reinforcing the importance of understanding the developmental pathways that govern tissue differentiation and reproductive competence.

Several contributions further examine how disturbances during embryonic and fetal development may contribute to congenital anomalies and long-term disease susceptibility. The relationship between placental pathology and congenital heart defects underscores the close developmental and functional interplay between the placenta and the fetal cardiovascular system, supporting the concept of a “heart–placental axis” during embryogenesis [2]. Similarly, congenital pulmonary airway malformation is examined through both clinical and developmental perspectives, illustrating how disruptions in early organogenesis can manifest as significant neonatal pathology [3].

The Special Issue also highlights the importance of maternal and intrauterine factors in shaping fetal and neonatal outcomes. Maternal vitamin D deficiency is associated with adverse effects on newborn health, emphasizing the critical contribution of maternal nutritional status to fetal development [4]. In parallel, the role of gut microbiota, inflammation, and probiotic supplementation in fetal growth restriction further illustrates the complex interactions between maternal physiology, placental function, and fetal growth dynamics [5]. These studies collectively reinforce the concept that fetal development is profoundly influenced by the maternal environment and that even subtle physiological imbalances may have long-term developmental consequences.

Neurodevelopmental outcomes also emerge as an important focus within this collection. Prenatal nutritional factors are examined in relation to neurodevelopmental disorders, highlighting how environmental and metabolic influences during critical developmental windows may contribute to alterations in brain development and later neurological function [6]. Complementing these observations, the investigation of β3-adrenoceptor expression in cord blood explores molecular adaptations to the hypoxic intrauterine environment, offering insight into fetal physiological regulation during development [7].

Importantly, this Special Issue extends beyond mechanistic developmental biology into the field of epidemiology and population health. A retrospective analysis from Bahrain investigates the prevalence and predictive factors of congenital anomalies, emphasizing the importance of epidemiological surveillance, risk stratification, and prenatal screening strategies in reducing disease burden and improving healthcare planning [8]. At the same time, the relationship between environmental stressors, catastrophic events, and the secondary sex ratio highlights how large-scale ecological and societal factors may influence reproductive and developmental outcomes at the population level [9].

Taken together, the contributions in this Special Issue illustrate that embryonic and fetal development must be understood through an integrated framework that spans molecular biology, stem cell research, maternal–fetal medicine, congenital pathology, and epidemiology, while reinforcing broader developmental frameworks connecting embryogenesis with human disease and translational medicine [10]. They collectively demonstrate how developmental processes are shaped by genetic, environmental, nutritional, physiological, and societal influences, while emphasizing the importance of interdisciplinary approaches for understanding, predicting, and ultimately preventing developmental disorders and adverse health outcomes.

## Abbreviations

The following abbreviations are used in this manuscript:

MSCs	Mesenchymal stem cells
